# Clinical Management of Herpes Simplex Virus Keratitis

**DOI:** 10.3390/diagnostics12102368

**Published:** 2022-09-29

**Authors:** Bisant A. Labib, DeGaulle I. Chigbu

**Affiliations:** Pennsylvania College of Optometry, Salus University, Elkins Park, PA 19027, USA

**Keywords:** herpes simplex virus, epithelial keratitis, stromal keratitis, antivirals, viral replication

## Abstract

Herpes simplex virus (HSV) keratitis is one of the leading causes of blindness worldwide. Additionally, up to 90% of the population in some countries is seropositive for HSV. HSV can cause a wide spectrum of ocular disease ranging from blepharitis to retinitis. Although the initial clinical expressions of HSV-1 and HSV-2 are similar, HSV-2 has been reported more frequently in association with recurrent HSV disease. Besides irreversible vision loss from keratitis, HSV also causes encephalitis and genital forms of the disease. Despite these statistics, there remains no vaccine against HSV. Current treatment therapies for related ocular diseases include the use of oral and topical antivirals and topical corticosteroids. While effective in many cases, they fail to address the latency and elimination of the virus, making it ineffective in addressing recurrences, a factor which increases the risk of vision loss. As such, there is a need for continued research of other potential therapeutic targets. This review utilized several published articles regarding the manifestations of HSV keratitis, antiviral immune responses to HSV infection, and clinical management of HSV keratitis. This review will summarize the current knowledge on the host–virus interaction in HSV infections, as well as highlighting the current and potential antiviral therapeutics.

## 1. Introduction Epidemiology, and Disease Burden

The herpes simplex virus (HSV), particularly HSV-1, is the leading cause of blindness due to infection in the developed world. It is estimated that approximately 50% of adults in the United States are seropositive for HSV, and up to 90% in regions such as Africa [1,2]. Globally, 67% of people under 50 years of age have been exposed to HSV1 and 11% to HSV 2 [3,4]. HSV related keratitis occurs in 149 per 100,000 in the developed world, and higher in less developed countries. New cases of HSV keratitis are estimated to be 18–25 per 100,000 and recurrences of 50% at 5 years and 60% or greater at 20 years [5,6]. Globally, it has been estimated that approximately 1.5 million cases of HSV related ocular infection occur every year, with 40,000 of those ending up with longstanding visual detriment [5].

The virus’s ability to spread through airborne droplets contributes to its high degree of transmissibility, often leading to blinding corneal ulcers. While HSV can affect any part of the eye, in the United States alone, 30,000 people suffer from recurrent corneal HSV involvement [7,8]. Besides the ocular sequelae of HSV-1, systemic manifestations also include encephalitis and genital herpes [9]. In addition to the risk of severe vision loss related to HSV keratitis, it impacts quality of life and poses a significant economic burden. A study in 2003 estimated a cost of 17.7 million dollars annually due to HSV keratitis [10]. This obviates the need for safe and effective treatment options, as there is no existing HSV vaccines at this time.

## 2. Host–Virus Interaction

HSV is an enveloped, double-stranded DNA virus that belongs to the Alphaherpesvirinae subfamily of the Herpesviridae family [11]. The structure of HSV-1 includes the viral envelope with glycoproteins, viral tegument, capsid, and DNA genome [12]. It has tropism for mucoepithelial cells and neurons. It causes lytic infection of epithelial cells and latent infection of most neurons. There is about 50% sequence homology between HSV-1 and HSV-2 [11,13,14].

### 2.1. Virology

Primary HSV infection is initiated by HSV glycoprotein B (gB), gC, gD, gH, and gL playing a role in HSV entry and fusion with the plasma membrane of host cells [15,16]. Glycoprotein B and glycoprotein C interact with heparan sulfate proteoglycans (HSPG) expressed on the surface of host cells to mediate the initial attachment of the HSV with the host cell. This results in a conformational change that triggers an interaction between gD and host cell receptors, such as nectin-1 expressed on neurons and herpes virus entry mediators (HVEM) expressed on epithelial cells [16,17]. This interaction triggers a conformational change in gD that mobilizes gH and gL to form the HSV multiglycoprotein complex, which results in the fusion of the viral envelope with the plasma membrane of the host cell, and subsequent endocytosis of HSV. Viral endocytosis is followed by viral uncoating which releases the nucleocapsid with tegument protein into the host cytoplasm [15,16,17]. Following uncoating of HSV, viral protein 16 (VP16) is released into the cytoplasm of host cells. VP16 is a structural protein that binds to host cell factor-1 (HCF) to form a VP16-HCF complex. It has been demonstrated that HCF plays a role in the trafficking of VP16 to the nucleus, and as such, it promotes the nuclear localization of VP16 as well as the assembly of the TRFC. Interaction between Oct-1 and the VP16-HCF complex results in the assembly of the transcription recognition factor complex (TRFC) [18]. The nuclear localization of VP16 as part of the transcription complex is necessary to activate the transcription of HSV immediate early genes [18]. Delivery of the viral DNA into the nucleus of the host cell is followed by production of HSV genes and genome replication [12,19]. Immediate early genes, early genes, and late genes are HSV-1 viral gene expressions that encode immediate early protein, early protein, and late proteins, respectively. HSV alpha genes are responsible for encoding immediate-early/alpha proteins such as infected cell protein 47 (ICP47), ICP0, and ICP4 which play a role in transactivation of early genes essential for HSV replication [19,20,21,22]. HSV early proteins are encoded by HSV early genes, and these early proteins play a role in HSV DNA genome replication [21,22]. Following genome replication, gamma genes are expressed to play a role in encoding structural and capsid proteins. The gamma or late proteins are responsible for assembling the capsid and membrane of the virus [22,23]. The capsid proteins are transported to the nucleus to be assembled into pro-capsids and filled with HSV DNA. The HSV nucleocapsid buds into the cytoplasm followed by synthesis of viral glycoprotein and viral envelope acquisition. Then, HSV is released by exocytosis or cell lysis [5]. The process of HSV replication in permissive corneal epithelial cells takes about 12 h, followed by local cell-to-cell spread [11,13,14,24] and syncytia formation [25,26]. The virus traverses the neuroepithelial gap to enter nearby sensory neurons. The nucleocapsid is transported to the neurons of the trigeminal ganglion to establish latent infection with intermittent reactivation of HSV [27]. During HSV-1 latency, there is no genome transcription; however, there is the generation of latency-associated transcripts (LAT) by HSV latently infected sensory neurons, which are required to maintain latency [28]. LAT is required to inhibit HSV lytic gene expression as well as suppressing the apoptosis of HSV infected sensory neurons [29] via preventing the effector function of Granzyme B secreted by trigeminal ganglion CD8^+^T cells [27,30]. Cell-mediated immunity against HSV-1 is responsible for inducing immune pressure on HSV, which drives the virus to latently infect neurons of the trigeminal ganglion. CD8^+^T cells are required to prevent reactivation of latent HSV in the neurons of the trigeminal ganglion. Th1 cells mediate type 1 immunity against HSV-1 [28]. Reactivation of HSV infected neurons results in the non-destructive replication of HSV in latently infected neurons, and the release of virions that reach the epithelial cells of the ocular surface via anterograde transport to cause HSV shedding and recurrent HSV keratitis [11,13,14].

### 2.2. Innate Immune Response in HSV Keratitis

During primary HSV infection of the cornea, viral glycopeptides and HSV DNA activate the innate and adaptive arms of the immune system. During HSV infection of the ocular surface, pattern recognition receptors (PRRs) are responsible for detecting the viral pathogen-associated molecular patterns (PAMPs) in infected cells, as well as damage-associated molecular patterns generated from damaged infected cells following HSV replication. The activation of these innate immune sensors, such as Toll-like receptors (TLRs) and retinoic acid inducible gene-I (RIG-I)-like receptors (RLRs), will induce downstream signaling transduction pathways, this culminates in the production of interferons and pro-inflammatory cytokines [31,32]. The innate immune response to infection of the ocular surface by HSV includes the generation of type I interferon (IFNα and IFNβ) by HSV infected epithelial cells of the ocular surface. Corneal epithelial cells express pattern recognition receptors, such as TLR2, TLR3, and TLR9 [33]. HSV-1 generates dsRNA during their replication in the corneal epithelium [34]. These dsRNA are released from HSV-infected, dying epithelial cells, and they are readily recognized by TLR3 expressed by corneal epithelial cells [35]. The interaction between dsRNA and TLR3 culminates in a downstream signaling cascade, in which, TIR-domain-containing adapter-inducing interferon-β (TRIF) is recruited and activated. The activated TRIF binds to tumor necrosis factor receptor associated factor 3 (TRAF3) and TRAF6. TRAF3 and TRAF6 engages and recruits TANK-binding kinase 1 (TBK1) and Inhibitory kappaB kinase (IKK), respectively. The recruited TBK1 and IKK phosphorylate interferon regulatory factor 3 (IRF3) and nuclear factor-kappaB (NF-κB), respectively. The activated IRF3 and NF-κB translocate to the nucleus where they stimulate genes required for coding interferons (type I IFN) and other cytokines [36,37,38,39,40,41]. Type I interferon induces an antiviral microenvironment, activates NK cells, and inhibits viral replication. The binding of type I IFN to its cognate receptor results in the recruitment and phosphorylation of signal transducer and activator of transcription 1 (STAT1) and STAT2 proteins. Binding of the phosphorylated STAT1 and STAT2 proteins to IFN-regulatory factor 9 (IRF9) yields the generation of interferon-stimulated gene factor 3 (ISGF3). In the nucleus of the HSV-infected cell, ISGF3 binds to interferon-stimulated response element (ISRE) on DNA, which triggers the transcription of interferon-stimulated genes (ISG). ISGs induce an antiviral response to control the HSV infection of the cornea [41,42,43,44,45]. Myeloid dendritic cells (DC) and plasmacytoid DC have a role to play in the immune response to HSV infection. Myeloid dendritic cells secrete IL-12 and IL-15, whereas plasmacytoid DC secrete type I interferons in response to interaction between HSV PAMPs and pattern recognition receptors on these dendritic cells [31,46,47]. The cellular innate immune response against HSV is mediated by NK cells, which secrete TNF-α and IFNγ that mediate the noncytolytic control of HSV infection [48]. Perforin and granzyme secreted by natural killer cells are cytolytic enzymes that destroy HSV-infected host cells [49]. There is a crosstalk between dendritic cells and natural killer cells when dendritic cells bind to Nkp30 receptors on NK cells [50]. IL-12 and IL-15 produced by myeloid dendritic cells activate NK cells, and activated NK cells secrete IFNγ and TNFα that reciprocally promote the antigen presenting capability of DC [46,47].

### 2.3. Adaptive Immune Response in HSV Keratitis

Myeloid dendritic cells migrate to the regional lymph node where they encounter naïve T cells in the paracortex area of the lymph node. In the paracortex area, myeloid DC present viral antigenic peptides complexed to MHC class II molecules to naïve CD4^+^T helper cells. These activated CD4^+^T helper cells undergo proliferation, and subsequently in response to IL-12 secreted by myeloid DCs differentiate into Th1 cells that secrete IFN-γ, IL-2, and TNFα. Th1 cells assist in priming the CD8^+^T cell response and activating myeloid DC via the action of IL-2 and IFNγ, respectively. IL-2 drives the proliferation of CD8^+^T cells. IFNγ enhances the antigen presenting capability of myeloid DC, which in turn secrete IL-12 that drives the differentiation of CD8^+^T cells into Cytotoxic T lymphocytes (CTL). Additionally, Th1 cell-derived IFNγ promotes the differentiation of HSV-specific B cells into HSV-specific IgG-secreting plasma cells. Because of the direct cell to cell transmission of HSV, it often escapes the neutralizing antibodies produced against HSV. As such, these antibodies have a lesser role in immune protection [31]. T cells are involved in the immunopathology of HSV keratitis. In HSV infection, HSV-specific CD8^+^T cells perform noncytolytic and cytolytic functions to mediate clearance of HSV and destruction of HSV-infected cells, respectively [51,52]. Cytolysis of HSV infected corneal epithelial cells is mediated by perforin and granzyme B secreted by CTL. Noncytolytic clearance of HSV is mediated by IFNγ, which favors the generation of antiviral microenvironments. In these antiviral microenvironments, viral replication is inhibited [53,54]. The cytolytic mechanism of HSV clearance involves the activity of Fas ligand, perforin, and granzyme expressed by HSV-specific CD8^+^T cells. The Fas/FasL-mediated clearance of HSV infected corneal cells is mediated by HSV-specific CD8^+^T cells that express Fas receptors which interact with FasL expressed on HSV infected corneal cells. This interaction induces the apoptosis of HSV infected corneal cells. The Fas-mediated apoptosis involves the activation of caspase-8 and caspase-9 and subsequent activation of downstream caspase-3, -6, and -7, which cause cell death [55,56]. Perforin and granzyme B released by HSV-specific CD8^+^T cells induce apoptosis of HSV infected corneal cells via granzyme B cleaving pro-caspase [57,58].

## 3. HSV Keratitis

Primary HSV infection of related anterior segment conditions, such as conjunctivitis or keratitis, is acquired through direct contact through mucous membranes. The virus, even following resolution, remains harbored in the trigeminal ganglion, where there is ophthalmic distribution. As such, recurrence is common; 40% of patients experience 2–5 relapses and 11% experience 6–15 relapses [59,60,61]. Although there is no definite gender or race predilection for HSV keratitis, some reports claim an increase in incidence for women, but a higher risk of recurrences in men [7].

Due to the recurrent nature of the infection, vision loss is common and typically occurs with stromal corneal involvement. HSV can manifest anywhere in the eye, but is most commonly presented in the cornea, specifically the epithelium as a dendritic keratitis. Once it invades the corneal stroma, it results in a more severe visual compromise and risk of recurrence [62]. It has been reported that over a 30-year period, 11% of patients end up with a best corrected visual acuity of 20/200 or worse [63]. In patients who do retain good visual acuity (VA) following HSV ocular infection, the quality of vision is often affected. This is due to aberrations from corneal scarring or induced astigmatism. Bilateral disease is also possible, though rare, and more severe [64].

### 3.1. Epithelial Keratitis

Epithelial keratitis from HSV infection presents variably from a punctate corneal keratitis to dendritic keratitis or geographic ulcer. Symptoms of epithelial keratitis manifest clinically as eye pain, redness, tearing, and foreign body sensation. Decreased corneal sensitivity is also a common manifestation, due to damage to corneal nerves, leading to neurotrophic keratopathy, further exacerbating the disease, and yielding visually damaging effects such as corneal perforation and melt [62]. Dendritic keratitis has a characteristic branching epithelial lesion with terminal end bulbs that harbor the live virus. As the disease progresses, the dendritic ulcer begins to coalesce and form geographic ulceration that is characterized by discrete flat edges (Table 1) [62].

Epithelial keratitis is typically mild and self-limiting, lasting from two to three weeks depending on severity and treatment. Non-pharmacological treatment approaches for HSV epithelial keratitis include debridement, which is used as a solitary approach or in conjunction with antiviral use to treat and prevent recurrences. Studies on efficacy are inconclusive and variable [70]. Some have shown that both antivirals and debridement can ease symptoms and shorten the course of the infection [70,71,72]. Amniotic membranes are also a potential treatment approach in patients with recurrent disease, in conjunction with antivirals, due to their anti-inflammatory effects [73]. Persistent epithelial keratitis is often due to poor compliance with antiviral administration or, rarely, from antiviral resistance. Another consideration for persistence is metaherpetic keratitis, following ulceration in the absence of active viral infection. The reason for this is neurotrophic in nature and can be managed by the use of topical lubricants, temporary tarsorrhaphy, therapeutic ptosis, amniotic membrane, topical autologous serum, or recombinant human nerve growth factor [65,74,75].

Diagnosis of epithelial keratitis is made through clinical examination with slit lamp biomicroscopy. The typical, dendritic lesion contains terminal end bulbs with swollen borders and intraepithelial cellular infiltration. The use of vital dyes can aid in identifying these lesions. Both lissamine green and rose Bengal staining enhance the appearance of the dendrite, facilitating diagnosis [76]. Yokogawa et al. utilized confocal microscopy to map out epithelial lesions and evaluate cellular changes such as the appearance of hyperreflective, irregular epithelial cells surrounded by multinucleated giant cells [77]. For less typical presentations that are not as readily identifiable on clinical presentation, polymerase chain reaction (PCR) has been used to confirm the diagnosis of epithelial HSK. Additional, more recent methods include tear collection to determine viral load and immunofluorescence antibody assay (IFA) to detect the viral antigen. PCR is the most sensitive method, and far superior to viral culture. However, this must be utilized prior to initiating antiviral treatment for best accuracy. It has been reported that using vital dyes may also interfere with PCR accuracy and inhibit HSV DNA detection [78].

### 3.2. Stromal Keratitis

Once the virus invades the stroma, it is deemed a stromal keratitis, which accounts for 20–48% of all ocular HSV infections [79]. Stromal keratitis is a result of a combination of the toxic, local effects of the virus as well as the host’s immunological response [64]. In this context, there is a higher risk of permanent vision loss due to deep corneal scarring and stromal neovascularization. Those who develop stromal disease are also at increased risk of recurrences, further increasing risk of vision loss (Table 1) [62]. 

The stromal forms of HSV keratitis can be classified as necrotizing and non-necrotizing, or disciform, keratitis. The necrotizing form of stromal keratitis appears as a fulminant stromal invasion of the virus, with or without epithelial ulceration. Due to the severity of the inflammatory response to the virus, patients who suffer from this form of stromal keratitis are at risk of corneal melt. The more common form is the non-necrotizing immune stromal keratitis. This is distinguished from the other form in that there is no necrosis, despite the presence of stromal inflammation, and a less likelihood of epithelial compromise. Clinically, this may manifest as a disciform lesion or ring, representing the deposition of the immune complex within the stroma. Edema is also commonly present in the inferior region of the corneal stroma, with or without the presence of keratic precipitates. Chronicity and recurrence of stromal disease leads to scarring, neovascularization, corneal thinning, and lipid deposition. HSV can penetrate further into the anterior segment, leading to endotheliitis and uveitis that is unique in that it often raises intraocular pressure and may present with iris atrophy [62,66,67].

Diagnosis of stromal HSV keratitis is primarily made through clinical examination and identification of the aforementioned characteristics. PCR is not as accurate or helpful in the diagnosis of the stromal form as it is for epithelial keratitis. Instead, enzyme-linked immunosorbent assay (ELISA) and viral cultures are sometimes utilized. ELISA works by detecting the virus from tears, but a caveat is that viral load often decreases after approximately 11 days, making time of diagnosis an important factor. Viral culture is considered the gold standard for diagnosis. With any method of diagnosis, timing and accuracy are critical to be able to promptly initiate treatment and prevent risk of permanent vision loss [76].

Although during primary infection, the virus undergoes replication within the corneal structures, it is later transported back through the ophthalmic nerve in a retrograde manner to the trigeminal ganglion where it causes latent infection. As such, HSV can be reactivated at any point, and is usually triggered by stress or immunosuppression. This leads to recurrences and further aggravation of the disease, as well as a greater likelihood to negatively impact vision [68]. 

Another complication of stromal keratitis and cause of vision loss is the development of stromal neovascularization. In order to accurately assess the degree and presence of neovascularization, multimodal imaging utilizing fluorescein and indocyanine green is superior to slit lamp examination alone, as this may underestimate the degree of the condition. Specific interventions include angiography guided fine needle diathermy of afferent feeder vessels, which is highly effective in cases where few feeder vessels are readily identified. This treatment is repeatable, with patients usually requiring approximately three or less sessions in total [69]. 

For multiple feeder vessels that are not as readily localized, anti-vascular endothelial growth factor (anti-VEGF) has been attempted with varying results. Its efficacy for corneal disease, especially neovascularization, which is already present, is questionable [64]. One study using both fine needle diathermy and anti-VEGF agent, bevacizumab, yielded no improvement in visual outcome despite minimizing the degree of neovascularization [80].

## 4. Clinical Management of HSV Keratitis 

### 4.1. Antivirals

Antivirals are the treatment of choice to combat HSV related ocular manifestations. Nucleoside analogues include acyclovir, ganciclovir, and trifluorothymidine, with acyclovir the most commonly used antiviral agent. Antivirals are available in oral and systemic forms. Acyclovir, valacyclovir, and famciclovir are Food and Drug Administration (FDA) approved for the treatment of HSV in their oral form, along with two topical antivirals: trifluridine and ganciclovir gel (Table 2) [55]. 

Acyclovir is a nucleoside analog that inhibits DNA polymerase and viral replication. It is a prodrug that enters the host and undergoes phosphorylation by various enzymes, such as thymidine kinase, to morph into its active form, acyclovir triphosphate [81]. Since it is phosphorylated by HSV thymidine kinase, mutations in thymidine kinase can lead to acyclovir resistance. Topical forms of acyclovir are available in Europe, but not in the United States at this time. It is the most commonly used antiviral due to its high affinity for HSV infected cells and an overall good safety profile [62]. 

Oral antiviral agents are effective in HSV keratitis. The Herpetic Eye Disease Study (HEDS) concluded that oral acyclovir was effective in reducing the risk of recurrence of stromal keratitis by 50% when used prophylactically. It was found that patients who were treated with oral acyclovir at a dose of 400 mg twice a day had a reduced risk of both epithelial and stromal keratitis by 45%. Caution should be exercised due to risk of developing antiviral resistance with prolonged use [82]. However, in active epithelial keratitis undergoing topical treatment already, the addition of oral acyclovir did not demonstrate further benefit [83]. Acyclovir is safe and well tolerated, making it an effective treatment option for HSV infection. Oral acyclovir possesses good bioavailability, but it is inversely proportional to its dosage amount. Absorption can be facilitated by the addition of a valine moiety. Though safe and well tolerated, acyclovir and other drugs in its class are subject to viral drug resistance and are not effective in latent disease [85].

Valaciclovir is a prodrug of acyclovir but has a greater bioavailability that is 3–5 times greater than acyclovir and comparable to intravenous forms. Famciclovir is a guanosine analog and is the least commonly used of the three [62]. 

Foscarnet is a pyrophosphate analogue DNA polymerase inhibitor. Because of its mechanism of action, it is often used in cases of acyclovir resistance because it is not metabolized by viral thymidine kinase. It does also, however, pose an issue of resistance attributed to mutations in viral DNA polymerase gene [64,84].

Trifluridine is a topical antiviral that is a pyrimidine nucleoside, used for epithelial keratitis. The frequency of administration limits its use, as it is prescribed up to nine times per day and may thus lead to epithelial toxicity. On the other hand, ganciclovir gel, a purine nucleoside, is dosed less at five times per day, with less propensity for epithelial toxicity. Both topical forms of antivirals appear to be comparable in efficacy, despite their differences [62]. 

### 4.2. Topical Corticosteroids and Immunological Agents

Stromal keratitis with trabeculitis and/or uveitis is complex in management, often necessitating the use of topical and oral antivirals, as well as topical corticosteroids. In order to penetrate the anterior chamber, acyclovir at a dose of 800 mg five times daily is warranted, in addition to the normal topical therapeutic dose [86]. To dampen the significant inflammatory effects of HSV, particularly in stromal keratitis, endotheliitis, trabeculitis, and uveitis, topical corticosteroids are the first line treatment. HEDS also demonstrated the efficacy of combining a topical antiviral, trifluorothymidine, with topical corticosteroids. Despite improvement in treatment efficacy with the addition of prednisolone in this study, half of treatment failures occurred within 6 weeks of discontinuing the steroid, suggesting the importance of a very slow and drawn-out taper. The ultimate endpoint visual acuity between the antiviral and placebo group versus antiviral plus corticosteroids was the same. As such, delaying or forgoing the use of corticosteroids does not bear any additional visual detriment. HEDS ultimately concluded that the use of topical corticosteroids reduced inflammation by 68% as compared to placebo [87]. Because of the known adverse effects of corticosteroids, particularly raised intraocular pressure and cataract, alternative treatments such as topical cyclosporine have offered some benefit in non-necrotizing infections [88,89]. Topical cyclosporine may be helpful for patients who respond poorly to corticosteroids. Additionally, interferon use in HSV can potentially mitigate the effects of viral replication and activity. While topical interferon α2B has shown promise in the treatment of HSV epithelial keratitis, it is not yet approved by the Food and Drug Administration (FDA) at this time (Table 2) [62].

### 4.3. Potential Drug Therapies and Targets

While the current treatment options are effective and viable approaches to HSV keratitis, there are limitations to efficacy as well as the problem of emerging antiviral resistance. Furthermore, current treatments do not address the virus in its latent form or offer full elimination of the infection. There is also no current treatment for the neurotrophic effects that HSV has on the corneal surface. 

A novel class of antivirals, known as helicase-primase inhibitors, works by preventing viral DNA synthesis. Examples of drugs in this class include protelivir and amenamevir, which have shown efficacy in herpes zoster and genital herpes. Both amanavir and pritelivir inhibit helicase-primase and decrease viral shedding and replication. SC93305 and BX795 may also potentially suppress viral replication (Table 3) [90,91]. This opened the door for further investigative therapies that target and inhibit viral helicase-primase and peptides [92,93]. One study suggested the use of BX795, which is a TANK-binding kinase 1 inhibitor, to block HSV-1 in infected cells [94]. Its impact on HSV and related ocular infections is not yet established and warrants further research [95].

Another drug, aganirsen, showed similar results, in that it significantly reduced corneal neovascularization, but did not show improvement in measured visual acuity. However, patients did report improvement in overall quality of life. Aganirsen works as an antisense oligonucleotide, inhibiting insulin receptor substrate-1 expression (Table 3) [96].

CRISPR-Cas9 was effective in blocking viral replication and showed promise in the removal of the virus from latency. This has shown promise in many of the herpes family viruses, including Epstein–Barr virus, human cytomegalovirus, and HSV-1 [97]. Recently, clustered regularly interspaced short palindromic repeats (CRISPR) was approved by the FDA for the treatment of diseases such as β-thalassemia, sickle cell disease, and Leber Congenital Amaurosis 10 (LCA-10) (ClinicalTials.gov: NCT04208529; NCT03745287; NCT03872479). Using this approach for infectious disease, both CRISPR and antiviral prodrugs were able to demonstrate viral clearance where latent HIV-1 infections were harbored in mice [98]. In another study specific to latent HSV-1 in mice, a targeted endonuclease using AAV did not show loss of viral genome or subsequent therapeutic effect [99]. Later on, however, utilizing an improved adeno-associated virus (AAV) vector and dual-meganuclease, there was found to be significant viral genome elimination and subsequent therapeutic effect (Table 3) [100].

Yin et al. documented the first in vivo study using mRNA-based CRISPR delivery to achieve a therapeutic effect against HSV-1 in human-derived corneas. Furthermore, using HSV-1-erasing lentiviral particle (HELP), evidence suggests that HSV-1 may be effectively eliminated from the trigeminal ganglion. HELP was administered in the same fashion as anti-VEGF when used to prevent stromal neovascularization in HSV keratitis [68].

Potential solutions include targeting the host cell receptors, such as 3-O-sulfated heparan sulfate and HSV envelope glycoproteins. Inhibition of viral entry utilizing these receptors can halt the spread of HSV and reduce the associated damaging effects (Table 3) [16].

Park et al. reported a peptide-acyclovir combination that was efficacious against HSV ocular infection. The use of G2 with acyclovir had greater antiviral activity. G2 is a cationic membrane penetrating peptide that binds to 3-O-sulfated heparan sulfate, which ultimately prevents HSV entry into cells. This was further demonstrated in a study using slow release of G2 through a contact lens in pig and human corneas. Other potential therapeutic targets include HSV glycoprotein gD and enzyme heparanase to prevent viral entry and spread (Table 3) [101]. 

Further anti-inflammatory and anti-angiogenic agents are under investigation for their efficacy in the detrimental effects of HSV keratitis. Azacytidine, which is FDA approved for the treatment of myelodysplastic syndrome, and retinoic acid have been shown to stabilize regulatory T cells and reduce inflammation in mice with HSV keratitis [113,114]. Other promising targets include immune modifying nanoparticle therapy and pigment epithelial-derived factor (PEDF) and docosahexaenoic acid (DHA), which can assist with preserving corneal sensitivity and reducing inflammation and neovascularization, respectively [115,116]. Lipid mediator resolvin D1 and microRNAs, particularly Mirl 55, can also ease stromal keratitis and neovascularization [117,118]. Mirl 132 inhibitory nanoparticles have shown evidence of reducing neovascularization in mouse models with HSV stromal keratitis [119].

In addition to VEGF, an Src kinase inhibitor molecule was shown to reduce the degree of HSV keratitis and subsequent corneal angiogenesis in mice models. Another molecule, Robo4, when administered, had a similar result, due to its role in anti-angiogenesis and VEGF signaling (Table 3) [102].

6-thioguanine (6-TG) is a thiopurine drug which is FDA approved as an anticancer drug specifically for acute lymphoblastic leukemia and other hematological malignancies. It works through the conversion of thioguanine deoxynucleotides into cellular DNA to kill cancer cells [103,104,105]. These drugs have also been used in the treatment of inflammatory bowel disease, which evidenced their mechanism of suppressing the small GTPase Rac1, yielding anti-inflammatory and immunosuppressive effects (Table 3) [104,106,107]. Chen et al. investigated the use of 6-TG in HSV ocular infection and found it to be more potent than both acyclovir and ganciclovir. When applied topically to the ocular surface, it alleviated the effects of HSV and, moreover, was effective in acyclovir resistant strains [105]. 

Harringtonine is a natural alkaloid and homolog which exhibits antitumor activities, giving for its use in leukemia. Its mechanism of action is that it blocks peptide bond formation and aminoacyl-tRNA binding, ultimately preventing protein synthesis (Table 3) [108]. More recently, its antiviral effects have been recognized, particularly against chikungunya virus [109], Singapore grouper iridovirus [110], varicella-zoster virus [111], and Zika virus [112]. Liu et al. demonstrated its efficacy against five HSV-1 strains, two of which were resistant to acyclovir. This was accomplished by harringtonine’s ability to suppress the herpes virus entry mediator expression, thus preventing viral entry of HSV-1 [9]. Finally, in 2021, Zhang et al. found that Ras-related C3 botulinum toxin substrate 1 could be a novel therapeutic target against HSV-1 [108].

## 5. Conclusions

While antivirals are a viable treatment for HSV keratitis, there continues to be profound vision loss due to HSV. As there is no vaccine at this time, there is a need for effective therapies that not only safely treat the active HSV infection but aim to prevent latent infection. Understanding the pathogenesis and virulence features of HSV can give rise to novel therapeutic targets to prevent viral entry, shedding, and replication.

## Figures and Tables

**Table 1:** Summary of corneal manifestations of HSV.

Corneal Location	Type	Presentation	Other Clinical Findings
**Epithelium**	Dendritic keratitis	Branching lesion with terminal end bulbs [62].	Punctate keratitis, Decreased corneal sensitivity, Neurotrophic keratitis or ulcer [62,65].
	Geographic ulcer	Coalesced dendrite with discrete flat edges [62].	
**Stroma**	Necrotizing	Fulminant stromal invasion of the virus, with or without epithelial ulceration [62,66,67].	Scarring, neovascularization, corneal thinning, and lipid deposition [68,69].
	Non-necrotizing/disciform	Disciform ring of virus; stromal inflammation without epithelial compromise [62,66,67].	

**Table 2:** Summary of current treatment for HSV keratitis.

Class	Drugs	Uses
Antivirals	Topical: trifluridine, ganciclovir gel [62] Oral: acyclovir, valacyclovir, famciclovir [62,81,82,83,84].	Treatment of epithelial keratitis, treatment of stromal keratitis and prophylactic use preventing recurrence in stromal keratitis [82,83,85].
Corticosteroids	Prednisolone	Stromal keratitis, endotheliitis, trabeculitis, and uveitis [86,87,88,89].

**Table 3:** Summary of potential therapeutic options for HSV keratitis.

Mechanism	Result
Helicase-primase inhibitors	Inhibit viral DNA synthesis [90,91,92,93].
BX795	TANK-binding kinase 1 inhibitor [94].
Aganirsen	Antisense oligonucleotide inhibiting insulin receptor substrate-1 expression [96].
CRISPR-Cas9	Blocks viral replication [97,98,99,100].
3-O-sulfated heparan sulfate	Prevents viral entry into host cell [16].
G2	Binds to 3-O-sulfated heparan sulfate to prevent viral entry [101].
Src kinase inhibitor molecule and Robo4	Reduced corneal angiogenesis [102].
6-thioguanine	Suppresses GTPase Rac1 causing anti-inflammatory and immunosuppressive effects [103,104,105,106,107].
Harringtonine	Blocks peptide bond formation and aminoacyl-tRNA binding and protein synthesis [108,109,110,111,112].

## Data Availability

No new data were created or analyzed in this study. Data sharing is not applicable to this article.
